# Effect of Lysine Supplementation in Low-Protein Diets on Nutrients Digestion, Growth Performance, Serum Biomarkers, and Production Performance of Female Blue Foxes (*Alopex lagopus*) in Fur-Growing Phase

**DOI:** 10.3390/ani15111559

**Published:** 2025-05-27

**Authors:** Yeye Geng, Xuezhuang Wu, Xiuhua Gao, Tietao Zhang, Qingkui Jiang

**Affiliations:** 1 Forestry College, Beihua University, Jilin 132013, China; beihuagengyeye@126.com; 2 Engineering Research Centre of Forestry Biotechnology of Jilin Province, Jilin 132013, China; 3 College of Animal Science, Anhui Science and Technology University, Chuzhou 233100, China; wuxuezhuang@126.com; 4 Feed Research Institute, Chinese Academy of Agricultural Sciences, Beijing 100081, China; gaoxiuhua@caas.cn; 5 Institute of Special Animal and Plant Sciences, Chinese Academy of Agricultural Sciences, Changchun 132109, China; 6 Public Health Research Institute, New Jersey Medical School, Rutgers Biomedical and Health Sciences, Rutgers, The State University of New Jersey, 225 Warren Street, Newark, NJ 07103, USA

**Keywords:** lysine, low-protein diets, amino acid, female blue foxes, fur-growing phase

## Abstract

This study evaluated the effects of dietary lysine supplementation in low-protein diets on nutrient digestibility, nitrogen metabolism, growth performance, serum biomarkers, and pelt quality in female blue foxes (*Alopex lagopus*) during the fur-growing period. A total of 105 18-week-old female blue foxes were randomly assigned to seven groups (n = 15 per group). The control group received a standard-protein diet (28% dry matter, DM), while six experimental groups were fed low-protein diets (26% DM) supplemented with 0%, 0.2%, 0.4%, 0.6%, 0.8%, and 1.0% lysine, corresponding to total lysine levels of 0.75%, 0.95%, 1.15%, 1.35%, 1.55%, and 1.75% DM, respectively. Lysine supplementation at 1.35% and 1.55% DM significantly improved the digestibility of ether extract and amino acids, including aspartic acid, glycine, methionine, isoleucine, and tyrosine (*p* < 0.05). Nitrogen retention increased accordingly, indicating enhanced dietary utilization (*p* < 0.05). Daily weight gain, particularly from day 15 to day 30, was significantly higher in 1.15–1.55% lysine groups compared to low-lysine groups (*p* < 0.05), achieving growth performance comparable to the control (*p* > 0.05). Serum total protein and albumin concentration were significantly improved with increasing lysine levels in low-protein groups (*p* < 0.01), aligning with those of the control group (*p* > 0.05). Furthermore, high lysine supplementation significantly improved pelt quality, as evidenced by the increased underfur length and decreased guard hair/underfur in 1.35–1.75% DM (*p* < 0.05). These findings suggest that lysine supplementation in low-protein diets supports nutrient utilization, growth performance, and metabolic health status while reducing dietary protein content. The optimal dietary lysine range is 1.15% to 1.55% DM (corresponding to 0.4% to 0.8% in air-dry basis), with 1.35% DM (corresponding to 0.6% in air-dry basis) identified as the most suitable level for balancing growth, nitrogen excretion, and pelt quality in fur-growing female blue foxes.

## Simple Summary

Feeding animals with less protein can reduce costs and environmental impact, but it may also affect their growth and health. This study looked at whether adding extra lysine, an essential nutrient, to a low-protein diet could support the health and fur quality of female blue foxes during their fur-growing season. One group of foxes received a normal-protein diet, while the others were given a slightly lower-protein diet with different levels of added lysine. The results showed that adding lysine improved how well the foxes digested nutrients and helped their bodies use protein more effectively. It also supported better growth, especially in the middle of the fur-growing period. Blood tests showed improved nutritional status, and the quality of the fur improved with longer underfur. The best balance of health, growth, and fur quality was seen when lysine made up about 1.35% of the diet. These findings suggest that carefully adding lysine to low-protein diets can help maintain animal health and fur value, while also reducing feed costs and nitrogen waste—supporting more sustainable farming practices in the fur industry.

## 1. Introduction

The commercial farming of blue fox (*Alopex lagopus*) is an important part of the fur industry. As domesticated carnivorous mammals, blue foxes require diets with a good proportion of protein feedstuffs of high biological values to support their growth, reproduction, and overall health [1]. This is particularly critical during the fur-growing phase of farmed blue foxes for several reasons. First, crude protein (CP) provides the necessary building blocks for the synthesis of keratin, the primary protein in fur. Adequate protein levels ensure optimal growth and development of the fur, leading to a denser, softer, and more luxurious pelt [2]. Second, sufficient protein supports general growth and body maintenance [3], ensuring that the foxes develop normally during the fur-growing phase.

Blue foxes’ high demand of protein-rich ingredients often leads to two issues. First, ingredients such as fishmeal and soybean meal are typically among the most expensive components of animal feed. Therefore, protein counts for a considerable proportion of production cost for fox ranches. In addition, excessive and/or imbalanced amino acids are presumably degraded and excreted by animals, primarily as nitrogen in urine and feces. This nitrogen can volatilize into the air as ammonia, contributing to air pollution and negatively affecting local air quality. It can also leach into water sources, causing nitrate contamination, which is harmful to aquatic ecosystems and can affect drinking water safety.

Modern animal nutrition emphasizes providing animals with the precise amount of protein and essential amino acids they need, rather than over-supplying protein. Reducing CP in feed not only lowers the overall cost of the feed formulation, but also decreases the amount of nitrogen excreted, helping to mitigate these environmental impacts. One way to achieve diet protein production is to supply low-protein diets with certain amino acids, which will balance the feed with the right amino acid profile and ensure better feed utilization, reducing the need for excess protein and improving the feed conversion ratio. This leads to more efficient growth with less feed, further lowering costs. Efforts have been made by others and us to decrease the level of dietary protein in order to provide inexpensive feed and still support animal performance and health, which has been the subject of research for several years [4,5,6,7].

A key factor limiting reduction in dietary CP is the requirement of lysine, which is a limiting amino acid in many common feed ingredients used in blue fox diets, such as plant-based proteins [8]. Ensuring sufficient lysine levels in the diet improves overall protein utilization, promoting efficient growth and development. Furthermore, lysine is essential for the synthesis of keratin, the structural protein that makes up fur. Adequate lysine intake ensures proper fur development, influencing both the density and quality of the pelt. We have previously shown that lysine supplementation in low-protein diets improves nutrients digestibility, growth performance, and healthy status of female blue foxes in the growing phase [6]. However, the optimal amount of lysine supplementation in low-protein diets during the fur-growing phase remains underexplored. The present study aimed to investigate the effect of low-protein diets with lysine supplementation on nutrient digestibility, nitrogen balance, growth performance, and serum parameters of female in the fur-growing phase.

## 2. Materials and Methods

### 2.1. Animals, Diets, and Management

The experiment was carried out at the Fur Animal Breeding Base of Institute of Special Animal and Plant Science, Chinese Academy of Agricultural Sciences (44.02° N, 126.15° E), in the northeast of China. The animals used in this work were managed according to the requirements of the national Experimental Animals Protection Law, with the experimental protocol being evaluated and approved by the Animal Care Committee of the Institute (NO.ISAPSAEC-2022-59MF).

A total of 105 healthy, 18-week-old female blue foxes with similar initial body weights were individually housed in cages placed under standard roofed sheds with open sides. At the beginning of the experiment, all animals were weighed and randomly distributed to seven groups, with 15 replicates per group. Each animal was considered an independent biological replicate to account for individual variability. The average initial body weights of the groups ranged from 4.54 to 4.69 kg, with no significant differences among groups. The control group received a standard diet containing 28% CP, while the six treatment groups were fed diets with a reduced CP level of 26%, supplemented with increasing levels of lysine at 0.75%, 0.95%, 1.15%, 1.35%, 1.55%, and 1.75%, respectively (Table 1).

Decreasing dietary protein levels (in the experiment from 261.10 to 281.00 g/kg dry matter, DM) in the treatment groups were compensated by increasing the level of dietary carbohydrates to ensure all the diets were isocaloric (14.51 MJ/kg to 14.48 MJ/kg). Dietary ether extract (EE) content remained constant within the range from 103.9 to 105.9 g/kg DM. To ensure acclimatization to the experimental conditions, all animals underwent a one-week adaptation period prior to the start of the trial. The experiment was conducted over a period of 75 days, during which blue foxes were provided the experimental diets twice daily *ad libitum* at 08:00 and 16:00 (Beijing time) with free access to drinking water. The ingredients and chemical composition of the diets are listed in Table 1, and amino acid content in Table 2.

### 2.2. Digestion Experiment

Nutrient digestibility was assessed as previously described [9]. Briefly, on day 70 of the trial, eight animals were randomly selected from each treatment group and transferred to individual metabolic cages designed to separately collect feces and urine. The digestibility trial lasted for three consecutive days, during which all excreta were collected daily. Feces were retrieved from collection plates beneath the cages, weighed, thoroughly homogenized, and sampled for laboratory analysis. To prevent ammonia loss from urine, 20 mL of 5% sulfuric acid was added to each urine collection bottle, and the urine trays were sprayed daily with a 20% citric acid solution. Fecal samples were dried at 65 °C for 24 h, then ground to pass through a 2 mm mesh sieve for subsequent chemical analysis.

### 2.3. Blood Sampling and Measurement

On the final day of the experiment, following the digestion and an overnight fast, blood samples were collected for the analysis of serum biochemical parameters. Serum samples were separated immediately by centrifugation at 2500× *g* for 5 min at 4 °C and stored at −80 °C until analysis of biochemical indicators. The total protein (TP), albumin (ALB), alkaline phosphatase (ALP), glutamic oxaloacetic transaminase (GOT), and glutamate pyruvate transaminase (GPT) were determined by an automatic biochemistry analyzer Hitachi 7020 (Hitachi, Ibaraki, Japan). Test kits were purchased from Nanjing Jiancheng Biochemical Corporation (Nanjing, China).

### 2.4. Chemical Analysis

Wet samples of diets were analyzed for DM and N [10].The freeze-dried samples of diets and feces were analyzed for CP, EE, and crude carbohydrate (CC). DM, CP (Kjeldahl-N × 6.25), EE, and CC after acid hydrolysis were determined according to standard procedures [10]. Amino acids in diets were analyzed by a L-8900 amino acid analyzer (Hitachi, Tokyo, Japan), as described by Ma, et al. [11]. Calculation of metabolic energy (ME) content and the proportional composition of ME were based on the digestibility coefficients achieved and the following values of ME: protein 18.8 MJ/kg, EE 39.8 MJ/kg, and carbohydrate 17.6 MJ/kg [12].

### 2.5. Growth Performance Evaluation

To evaluate growth performance, animals were weighed every 15 days using an electronic scale until the end of the experiment on day 75. The initial measurement at the start of the experiment was designated as day 0. Weighing was conducted at 7:30 MA after an overnight fast.

### 2.6. Pelt Quality

Pelt quality was determined as previously described [13]. Briefly, subcutaneous fat tissue adhering to the inner surface of the fur was carefully removed prior to measurement using a scalpel. Pelt qualities were determined after the pelts were fully dried and stretched. The pelt length was measured using a ruler from the base of the tail to the nose. Pelt weight was determined using a precision scale. Guard hair and underfur lengths were measured separately from the skin surface to the tip using a ruler, with multiple measurements taken from three body regions (back, sides, and belly) to calculate the mean length. The guard hair-to-underfur ratio was determined based on these mean values. Pelts from n = 15 animals per group were analyzed.

### 2.7. Statistical Analysis

Statistical analyses were carried out using GraphPad Prism (version 6.01, GraphPad Software, San Diego, CA, USA). Each animal served as an experimental unit. Differences among groups were evaluated using one-way ANOVA procedure. When significant differences were detected, Tukey’s multiple comparisons test was used to compare group means. Data were represented as mean ± SD. A *p* < 0.05 was considered statistically significant.

## 3. Results

### 3.1. Nutrients Digestibility

Table 3 shows the effect of lysine supplementation at varying levels (0.75%, 0.95%, 1.15%, 1.35%, 1.55%, and 1.75%) in a low-protein diet on the apparent digestibility of DM, CP, and EE in comparison to a control diet with common dietary protein content. The daily DM intake, DM output, and apparent digestibility of DM (%) across all treatment groups, including the control, remained consistent, suggesting that lysine supplementation did not significantly influence these indices. CP digestibility followed a similar pattern; with a slight upward trend with increasing lysine levels, no statistically significant differences were observed across the treatment groups. EE digestibility, however, showed a significant response to lysine supplementation with lysine supplementation. The 1.35% and 1.55% lysine group exhibited significantly higher EE digestibility (88.03% and 88.85%, respectively) compared to the 0.75% lysine (85.29%) (*p* < 0.05), indicating increased dietary lysine levels improved fat digestibility.

### 3.2. Amino Acid Digestibility

Table 4 shows lysine supplementation (0.75%, 0.95%, 1.15%, 1.35%, 1.55%, and 1.75%) in a low-protein diet influenced the apparent digestibility of various amino acids. The low-protein diet supplemented with low levels of lysine exhibits the poorest digestibility of amino acids. Of note, digestibility of various amino acids was increased in higher lysine level groups comparing to the control group, despite the low protein content. For instance, the 1.35% and 1.55% lysine groups showed maximum of 62.29% and 61.96% serine digestibility, increased from 53.65% in the control (*p* < 0.05). Digestibility of glutamic acid also improved with 1.35% lysine supplementation compared to the control (68.89% vs. 58.70%). Methionine digestibility increased significantly at higher lysine levels, reaching 92.51% at 1.15% lysine and remaining elevated at higher concentrations (*p* < 0.05). Tyrosine and phenylalanine digestibility followed a similar trend, with high lysine levels (1.15%, 1.35%, 1.55%, and 1.75%) showing increased digestibility in comparison to the low lysine and the control groups (*p* < 0.05). Interestingly, lysine digestibility itself was significantly improved by supplementation, reaching 82.33% at 1.35% lysine (*p* < 0.05), compared to 56.28% in the control.

### 3.3. Nitrogen Metabolism

Table 5 summarizes the effects of lysine supplementation in low-protein diets on nitrogen metabolism in blue foxes during the fur-growing stage. Nitrogen intake significantly increased with lysine levels, peaking at 17.52 g/d in the 1.75% group (*p* < 0.01). Although no significant differences were observed in fecal and urine nitrogen among groups, nitrogen retention, which reflects the balance between nitrogen intake and excretion (via feces and urine), was improved with higher lysine levels, reaching a maximum of 4.57 g/d in the 1.75% group (*p* = 0.04), indicating higher lysine level improved nitrogen utilization.

### 3.4. Growth Performance

Table 6 illustrates the effects of lysine supplementation in low-protein diets on the growth performance of blue foxes during the fur-growing stage. Body weight exhibited an increasing trend with higher lysine levels during the later stage of the trial, approaching statistical significance at both Day 60 (*p* = 0.08) and Day 75 (*p* = 0.07). By Day 75, foxes receiving 1.15–1.75% lysine had comparable body weights, whereas those in the low-lysine group (0.75%) consistently showed the lowest body weights (*p* = 0.07), suggesting a positive association between lysine intake and growth performance. In addition, significant improvements in daily weight gain were observed in the early stages (Days 0–30) with lysine supplementation, particularly in the 1.15% lysine group, which showed the highest gains during both Days 0–15 (49.12 g/L) (*p* = 0.02) and Days 16–30 (47.19 g/L) (*p* = 0.03). No significant differences were observed in later stages (Days 31–75). These results suggest that moderate-to-high lysine levels may enhance early growth performance in the fur-growing stage.

### 3.5. Serum Biomarkers

Table 7 summarizes the effects of lysine supplementation in low-protein diets on serum biomarkers of blue foxes during the fur-growing stage. TP levels significantly increased with lysine supplementation, with the highest levels observed in the 1.15–1.75% lysine groups (61.41 to 67.43 g/L), which are comparable to the control group (67.85 g/L) but higher than the low lysine-supplementation group (42.64 g/L in the 0.75% group and 44.1 g/L in the 0.95% group) (*p* < 0.01). ALB levels were significantly higher in the 1.75% lysine groups (38.73 g/L), which is similar to the control (38.29 g/L) but higher than the rest of the lysine groups (35.04 to 37.90 g/L) (*p* < 0.01). The ALP level in the 1.35% lysine group was comparable to that of the control group with a regular protein level and significantly higher than those in the low-lysine groups (0.75% and 0.95%) (*p* < 0.01). The GOT level was significantly elevated only in the 1.75% group (*p* < 0.01), while GPT levels showed no consistent trend across treatments, with fluctuations noted among groups. These findings indicate that lysine supplementation significantly enhances TP and ALB levels, while other serum biomarkers remain largely unaffected.

### 3.6. Pelt Quality

Table 8 highlights the effects of lysine supplementation in low-protein diets on pelt quality of blue foxes during the fur-growing stage. Pelt length and weight showed no significant differences among treatments, with lengths ranging from 94.50 cm to 99.88 cm and weights from 611.7 g to 660.8 g. The low-lysine group exhibited significantly longer guard hair (6.74 mm) compared to the medium- and high-lysine groups (6.22–6.28 mm in 1.35–1.75% lysine groups) and the control group (6.10 mm) (*p* < 0.01). In contrast, underfur length was significantly greater in the 1.35–1.75% lysine groups (5.38–5.47 mm) than in low-lysine groups (5.08–5.27 mm in 0.75–1.15% lysine groups) and the control (5.25 mm) (*p* < 0.01). As a result, the guard hair-to-underfur ratio significantly decreased with lysine supplementation, with the lowest ratio observed in the 1.35–1.75% lysine groups (1.15 to 1.16) compared to the control (1.32) (*p* < 0.01). These results suggest that lysine supplementation enhances underfur growth and reduces the guard hair-to-underfur ratio, contributing to improved fur quality.

## 4. Discussion

### 4.1. Feed Intake and Nutrients Digestibility

Low-protein diets supplemented with appropriate amino acids promote the metabolic value and growth performance of poultry, pigs, dogs, and mink [14,15,16,17,18]. In the current study, lysine supplementation had minimal effects on feed intake and the digestibility of DM and CP. Compared to the control group with regular protein level, EE digestibility at lysine levels of 1.35–1.55% was higher than that observed in the low-lysine group (0.75% and 0.95%), indicating that lysine supplementation enhances dietary fat utilization. Similarly, suitable dietary digestible lysine levels significantly improve crude fat digestibility in Yunan black pigs during the 60–90 kg growth stage [19]. These improvements in fat digestibility may be attributed to lysine’s role in carnitine synthesis [20], which is essential for converting fatty acids into energy through fatty acid oxidation [21]. Increased energy production efficiency could contribute to improved growth performance and fur quality in the fur-growing stage of blue foxes. These findings suggest that low-protein diets with optimized lysine levels can achieve comparable nutrient utilization as regular protein diets while potentially reducing dietary costs.

### 4.2. Amino Acid Digestibility

High dietary lysine levels significantly enhanced the digestibility of most amino acids, including lysine itself, compared to the low-lysine groups. Improvements were most pronounced for essential amino acids such as lysine, methionine, and leucine, which are critical for protein synthesis and growth. The enhanced digestibility of non-essential amino acids like glycine and proline further supports the role of lysine in optimizing overall amino acid metabolism. The results emphasize lysine’s importance in improving amino acid bioavailability and utilization, which is crucial for sustaining growth and fur development. The optimal effects observed at lysine levels of 1.35% and 1.55% demonstrate that low-protein diets with appropriate lysine supplementation can maintain efficient amino acids utilization comparable to regular protein diets.

### 4.3. N Retention

Lysine supplementation in a low-protein diet significantly influenced nitrogen metabolism in blue foxes. Compared to the 0.75% lysine group, nitrogen intake declined with increasing lysine levels up to 1.55%, followed by an increase in the 1.75% group, suggesting a non-linear response to dietary lysine supplementation. This pattern suggests that higher lysine levels may enhance dietary protein utilization, which is consistent with findings reported in rabbits [22]. Fecal nitrogen output showed no consistent trend, and urine nitrogen levels remained relatively stable across groups, indicating that lysine did not significantly alter nitrogen excretion pathways. Notably, nitrogen retention improved significantly in the higher lysine-supplemented groups (1.35–1.75%), highlighting enhanced protein retention. Dietary amino acid levels have been shown to affect nitrogen retention [23,24], especially in animals fed with low-protein diets [25]. We have previously reported that dietary lysine levels significantly influence N retention in blue foxes in the growing phase [6]. Our present study further contributes to the understanding that lysine plays a pivotal role in optimizing protein metabolism by improving nitrogen utilization efficiency. These findings suggest that low-protein diets with optimized lysine levels can potentially reduce nitrogen excretion, offering an environmentally sustainable approach [26].

### 4.4. Growth Performance and Pelt Quality

Lysine supplementation had a pronounced effect on the growth performance of blue foxes. While the low-lysine group (0.75%) showed decreased body weight, groups supplemented with higher lysine had comparable body weight to the control group with a regular protein level. The most notable gains were observed between 1.15% and 1.75% lysine levels. Daily weight gain was significantly higher during the initial and mid-experimental phases in lysine-supplemented groups, indicating that lysine supports rapid growth during these critical periods. These improvements likely result from enhanced protein synthesis facilitated by lysine’s role in amino acid metabolism [27]. However, the plateau observed at higher lysine levels suggests a potential ceiling effect, where further increases in lysine may not yield additional growth benefits.

Lysine supplementation also benefited pelt quality metrics. Notably, underfur length increased significantly with lysine levels between 1.15% and 1.55%, consistent with the growth performance results. Meanwhile, the guard hair-to-underfur ratio decreased, indicating a denser and finer fur coat. These improvements are critical for the fur industry, as they enhance the commercial value of the pelt [28]. The results align with lysine’s known role in supporting keratin synthesis, a key component of hair and fur [29,30], and demonstrated that lysine supplementation can enhance pelt quality in blue foxes of the fur-growing phase. These findings further highlight the potential of low-protein diets with optimized lysine levels to maintain production performance and pelt quality comparable to regular protein diets.

### 4.5. Serum Parameters

Serum parameters, such as TP, ALB, ALP, GOT, and GPT, are important indicators of protein metabolism and liver function. As the liver performs key tasks related to protein metabolism [31], nutrition status, especially protein and amino acids content, can influence hepatic functions [32]. In our study, lysine supplementation markedly improved serum biomarkers indicative of protein metabolism and liver function in low-protein diets. TP and ALB levels increased significantly with lysine supplementation, particularly at higher levels, reflecting improved nutritional status and protein turnover. In addition, the increased ALP and decreased GOT indicated liver damage in the low lysine groups [33]. While changes in GPT were minimal, the alterations in TP, ALB, ALP, and GOT suggest that lysine positively impacts overall protein metabolism and supports liver function. These findings underscore lysine’s role in maintaining metabolic health during periods of fur growth, aligning with the potential of low-protein diets to sustain health and production performance comparable to regular protein diets.

## 5. Conclusions

The findings demonstrate that lysine supplementation in low-protein diets has the potential to reduce dietary costs and environmental impact by lowering nitrogen excretion while maintaining comparable health status and production performance to regular protein diets. Our results indicate that optimal lysine levels for low-protein diets ranging from 1.15% to 1.55% DM (corresponding to 0.4% to 0.8% in air-dry basis), with 1.35% of DM (corresponding to 0.6% in air-dry basis), identified as the most suitable level for balancing growth, nitrogen excretion, growth performance, and pelt quality in fur-growing female blue foxes. These findings highlight the critical role of lysine in optimizing nutrient utilization and supporting fur development, offering a cost-effective and environmentally sustainable alternative to high-protein diets.

## Figures and Tables

**Table 1:** Composition and nutrient levels of the experimental diets (air-dry basis, %).

Items	Basal Diet (0.75% Lysine)	Control Diet
Ingredients		
Extruded corn	47.90	45.00
Soybean meal	15.70	15.00
Bone and meat meal	18.00	18.00
Corn germ meal	5.00	6.40
Fish meal	5.00	7.00
Soybean oil	7.00	7.00
Salt	0.30	0.30
Premix ^(1)^	1.00	1.00
Lys	0.00	0.20
Met	0.10	0.10
Total	100.00	100.00
Nutrient levels ^(2)^		
CP (%)	26.11	28.10
EE (%)	10.39	10.59
Ash	8.53	8.83
CC%	52.31	52.01
Lys (%)	0.75	0.99
Met (%)	0.89	0.89
ME (MJ/kg)	14.51	14.48
% of ME		
CP	21.52	23.48
EE	34.16	34.43
CC	44.32	42.09

Notes: ^(1)^ The premix contained per kg: Vitamin A 1,000,000 IU; Vitamin D_3_ 200,000 IU; Vitamin E 6000 IU; Vitamin B_1_ 600 mg; Vitamin B_2_ 800 mg; Vitamin B_6_ 300 mg; Vitamin B_12_ 10 mg; Vitamin K_3_ 100 mg; Vitamin C 40,000 mg; Nicotinic acid 4000 mg; Pantothenic acid 1200 mg; Alkaloid 20 mg; Folic acid 80 mg; Choline 30,000 mg; Fe 8200 mg; Cu 800 mg; Mn 1200 mg; Zn 5200 mg; I 50 mg; Se 20 mg; Co 50 mg. ^(2)^ Results in % of DM, except energy density (ME), in MJ/kg DM. Energy distribution as % of ME. Values of CP, EE, ash were measured, and others were calculated values.

**Table 2:** Amino acids content of experimental diets (% dry mater).

Items	Basal Diet (0.75% Lysine)	Control Diet
Aspartic acid	1.25	1.33
Threonine	0.62	0.66
Serine	0.76	0.80
Glutamic acid	2.44	2.54
Glycine	0.81	0.86
Alanine	1.08	1.12
Valine	0.70	0.74
Methionine	0.93	0.94
Isoleucine	0.64	0.69
Leucine	1.57	1.68
Tyrosine	0.52	0.56
Phenylalanine	0.80	0.85
Lysine	0.75	0.99
Histidine	0.39	0.41
Arginine	1.03	1.07
Proline	0.77	0.79

**Table 3:** Effect of lysine supplementation in low-protein diets on feed intake and nutrients digestibility of blue foxes in fur-growing stage.

Items	Lysine						Control	*p* Value
	0.75%	0.95%	1.15%	1.35%	1.55%	1.75%		
DM intake (g/d)	387.42 ± 1.16	387.55 ± 1.52	385.71 ± 3.75	386.92 ± 0.67	387.24 ± 0.49	387.88 ± 0.55	385.03 ± 2.28	0.13
DM output (g/d)	135.85 ± 13.58	134.10 ± 8.49	129.36 ± 8.35	134.48 ± 6.70	129.43 ± 11.86	126.67 ± 10.80	125.94 ± 7.79	0.37
DM (%)	64.93 ± 3.55	65.40 ± 2.20	66.46 ± 2.18	65.24 ± 1.76	66.57 ± 3.07	67.34 ± 2.78	67.30 ± 1.86	0.41
CP (%)	62.23 ± 4.82	63.10 ± 2.84	66.41 ± 5.02	66.41 ± 4.44	67.12 ± 4.79	65.15 ± 3.98	65.57 ± 4.25	0.22
EE (%)	85.29 ± 3.01 ^a^	87.47 ± 2.66 ^ab^	86.37 ± 2.87 ^ab^	88.03 ± 0.81 ^b^	88.85 ± 1.49 ^b^	86.48 ± 2.45 ^ab^	88.97 ± 0.91 ^b^	0.03

Notes: DM: dry matter; CP: crude protein; EE: ether extract. Differences among groups were determined using the one-way ANOVA procedure. Means with different lowercase superscripts differ significantly at *p* < 0.05.

**Table 4:** Effect of lysine supplementation in low-protein diets on amino acids digestibility of blue foxes in fur-growing stage.

Items	Lysine						Control	*p* Value
	0.75%	0.95%	1.15%	1.35%	1.55%	1.75%		
Aspartic acid	41.63 ± 5.61 ^a^	41.33 ± 2.74 ^a^	52.30 ± 5.62 ^b^	58.69 ± 7.75 ^b^	58.04 ± 7.60 ^b^	60.36 ± 3.67 ^b^	57.10 ± 8.56 ^b^	<0.01
Threonine	45.42 ± 7.47 ^a^	45.23 ± 6.11 ^a^	49.20 ± 7.40 ^ab^	57.96 ± 6.64 ^c^	58.16 ± 5.32 ^c^	57.75 ± 3.45 ^c^	54.51 ± 2.88 ^bc^	<0.01
Serine	49.75 ± 8.43 ^a^	51.67 ± 4.01 ^a^	55.27 ± 4.36 ^abc^	62.29 ± 4.49 ^c^	61.96 ± 5.33 ^c^	60.57 ± 2.64 ^bc^	53.65 ± 10.19 ^ab^	<0.01
Glutamic acid	53.19 ± 6.50 ^ab^	51.68 ± 3.43 ^a^	56.75 ± 7.38 ^abc^	68.89 ± 12.78 ^d^	63.64 ± 6.35 ^cd^	61.80 ± 2.59 ^cd^	58.70 ± 2.96 ^abc^	<0.01
Glycine	23.84 ± 7.77 ^a^	25.74 ± 6.59 ^a^	34.80 ± 10.07 ^b^	39.28 ± 8.22 ^bc^	39.50 ± 9.75 ^bc^	46.44 ± 1.40 ^c^	40.19 ± 6.44 ^bc^	<0.01
Alanine	51.09 ± 8.45 ^ab^	48.34 ± 10.21 ^a^	48.53 ± 9.74 ^a^	57.77 ± 7.13 ^ab^	60.10 ± 5.54 ^b^	56.25 ± 2.63 ^ab^	57.55 ± 3.70 ^ab^	0.03
Valine	28.00 ± 6.53 ^a^	32.40 ± 1.97 ^ab^	49.74 ± 5.79 ^c^	50.58 ± 5.75 ^c^	52.04 ± 8.22 ^c^	51.87 ± 3.89 ^c^	43.83 ± 4.59 ^bc^	<0.01
Methionine	85.54 ± 1.49 ^a^	86.43 ± 2.22 ^ab^	92.51 ± 1.42 ^c^	91.47 ± 1.75 ^c^	90.96 ± 1.38 ^c^	91.22 ± 1.19 ^c^	87.63 ± 1.79 ^b^	<0.01
Isoleucine	55.96 ± 5.54 ^ab^	53.76 ± 4.55 ^a^	60.53 ± 5.39 ^bc^	67.35 ± 6.48 ^d^	67.26 ± 5.44 ^d^	66.97 ± 2.25 ^d^	64.90 ± 2.36 ^cd^	<0.01
Leucine	68.07 ± 4.53 ^ab^	65.11 ± 5.64 ^a^	67.16 ± 4.80 ^ab^	73.81 ± 5.15 ^bc^	73.64 ± 4.87 ^bc^	72.04 ± 2.45 ^abc^	76.03 ± 9.58 ^c^	0.02
Tyrosine	65.05 ± 3.99 ^a^	61.92 ± 4.23 ^a^	73.80 ± 2.49 ^b^	78.53 ± 4.36 ^b^	75.16 ± 4.52 ^b^	78.39 ± 3.07 ^b^	65.72 ± 3.64 ^a^	<0.01
Phenylalanine	21.31 ± 6.35 ^a^	25.99 ± 7.15 ^a^	50.23 ± 5.57 ^c^	53.88 ± 6.19 ^c^	57.41 ± 6.11 ^c^	54.35 ± 6.69 ^c^	35.97 ± 5.44 ^b^	<0.01
Lysine	56.28 ± 4.76 ^a^	63.07 ± 1.93 ^b^	73.17 ± 2.92 ^d^	79.91 ± 2.79 ^e^	82.33 ± 3.02 ^e^	68.14 ± 3.82 ^c^	56.83 ± 4.18 ^a^	<0.01
Histidine	54.35 ± 7.08 ^a^	53.22 ± 3.17 ^a^	56.30 ± 5.32 ^ab^	61.93 ± 3.71 ^bc^	62.68 ± 7.48 ^bc^	64.05 ± 5.17 ^c^	64.72 ± 3.79 ^c^	<0.01
Arginine	74.78 ± 4.72 ^a^	73.48 ± 2.89 ^a^	75.54 ± 2.92 ^a^	78.89 ± 2.84 ^b^	77.87 ± 3.08 ^b^	77.83 ± 2.15 ^b^	78.49 ± 2.63 ^b^	0.03
Proline	44.98 ± 8.25 ^ab^	41.76 ± 8.95 ^a^	50.94 ± 9.12 ^abc^	64.00 ± 7.51 ^c^	62.65 ± 7.89 ^bc^	59.70 ± 10.03 ^bc^	60.18 ± 9.27 ^bc^	0.04

Notes: Differences among groups were determined using the one-way ANOVA procedure. Means with different lowercase superscripts differ significantly at *p* < 0.05.

**Table 5:** Effect of lysine supplementation in low-protein diets on nitrogen metabolism of blue foxes in fur-growing stage.

Items	Lysine						Control	*p* Value
	0.75%	0.95%	1.15%	1.35%	1.55%	1.75%		
N intake (g/d)	16.90 ± 0.05 ^a^	16.35 ± 0.06 ^b^	16.32 ± 0.16 ^b^	16.57 ± 0.16 ^cd^	16.71 ± 0.02 ^d^	17.52 ± 0.03 ^e^	16.47 ± 0.15 ^bc^	<0.01
Fecal nitrogen (g/d)	5.82 ± 0.72	6.03 ± 0.45	6.16 ± 0.76	5.36 ± 0.65	5.49 ± 0.80	6.19 ± 0.60	5.68 ± 0.36	0.12
Urine nitrogen (g/d)	7.10 ± 0.61	7.15 ± 0.45	6.96 ± 0.29	6.94 ± 0.31	6.90 ± 0.63	6.76 ± 0.57	6.72 ± 0.42	0.60
N retention (g/d)	3.98 ± 1.13 ^ab^	3.17 ± 0.73 ^a^	3.20 ± 1.00 ^a^	4.26 ± 0.50 ^ab^	4.31 ± 1.26 ^ab^	4.57 ± 1.14 ^b^	4.08 ± 0.47 ^ab^	0.04

Notes: Differences among groups were determined using the one-way ANOVA procedure. Means with different lowercase superscripts differ significantly at *p* < 0.05.

**Table 6:** Effect of lysine supplementation in low-protein diets on growth performance of blue foxes in fur-growing stage.

Items	Lysine						Control	*p* Value
	0.75%	0.95%	1.15%	1.35%	1.55%	1.75%		
Body weight (kg)								
Day 0	4.54 ± 0.32	4.69 ± 0.34	4.62 ± 0.32	4.67 ± 0.32	4.60 ± 0.45	4.62 ± 0.33	4.59 ± 0.29	0.10
Day 15	5.0 ± 0.38	5.22 ± 0.33	5.36 ± 0.38	5.34 ± 0.40	5.24 ± 0.51	5.29 ± 0.36	5.10 ± 0.43	0.72
Day 30	5.51 ± 0.39	5.84 ± 0.39	6.07 ± 0.46	6.09 ± 0.45	5.93 ± 0.45	6.00 ± 0.32	5.79 ± 0.52	0.22
Day 45	5.82 ± 0.48	6.23 ± 0.46	6.40 ± 0.52	6.46 ± 0.52	6.35 ± 0.46	6.39 ± 0.33	6.13 ± 0.47	0.26
Day 60	6.20 ± 0.58	6.73 ± 0.57	6.98 ± 0.64	6.98 ± 0.57	6.93 ± 0.45	6.96 ± 0.39	6.57 ± 0.43	0.08
Day 75	6.65 ± 0.61	7.14 ± 0.65	7.49 ± 0.71	7.45 ± 0.63	7.42 ± 0.60	7.50 ± 0.50	7.09 ± 0.29	0.07
Daily weight gain (g)								
D0–D15	33.38 ± 20.50 ^a^	35.74 ± 12.60 ^ab^	49.12 ± 11.92 ^b^	43.83 ± 15.30 ^ab^	42.11 ± 8.55 ^ab^	44.72 ± 13.63 ^ab^	33.54 ± 11.96 ^a^	0.02
D16–D30	31.18 ± 17.86 ^a^	41.33 ± 12.04 ^ab^	47.19 ± 10.49 ^b^	48.71 ± 8.00 ^b^	46.28 ± 15.75 ^ab^	47.33 ± 11.96 ^b^	46.36 ± 19.61 ^ab^	0.03
D31–D45	21.13 ± 12.75	25.95 ± 7.38	22.00 ± 7.75	26.19 ± 12.35	28.22 ± 9.63	26.11 ± 9.30	22.56 ± 8.72	0.48
D46–D60	25.44 ± 11.82	32.92 ± 10.04	38.57 ± 11.85	34.86 ± 8.70	38.11 ± 9.42	37.56 ± 13.37	29.13 ± 14.62	0.03
D61–D75	29.64 ± 12.46	27.18 ± 16.46	34.00 ± 20.20	33.05 ± 15.66	33.22 ± 15.13	36.00 ± 15.84	35.08 ± 15.15	0.81

Notes: Differences among groups were determined using the one-way ANOVA procedure. Means with different lowercase superscripts differ significantly at *p* < 0.05.

**Table 7:** Effect of lysine supplementation in low-protein diets on serum biomarkers of blue foxes in fur-growing stage.

Items	Lysine						Control	*p* Value
	0.75%	0.95%	1.15%	1.35%	1.55%	1.75%		
TP (g/L)	42.64 ± 8.69 ^a^	44.1 ± 3.11 ^a^	63.95 ± 6.12 ^b^	65.62 ± 5.36 ^b^	61.41 ± 2.99 ^b^	67.43 ± 12.28 ^b^	67.85 ± 12.56 ^b^	<0.01
ALB (g/L)	35.56 ± 1.36 ^ac^	35.04 ± 1.72 ^c^	35.85 ± 1.21 ^ac^	36.50 ± 1.38 ^ac^	37.90 ± 1.19 ^a^	38.73 ± 1.84 ^b^	38.29 ± 1.39 ^bc^	<0.01
ALP (U/L)	104.40 ± 39.19 ^a^	103.90 ± 10.04 ^a^	77.18 ± 14.65 ^ab^	57.77 ± 17.86 ^b^	74.9 ± 4.86 ^ab^	73.29 ± 9.36 ^ab^	58.18 ± 7.23 ^b^	<0.01
GOT (U/L)	97.98 ± 13.41 ^a^	137.80 ± 20.02 ^a^	149.70 ± 44.61 ^a^	145.00 ± 25.98 ^a^	158.40 ± 60.51 ^a^	243.10 ± 41.15 ^b^	111.5 ± 25.31 ^a^	<0.01
GPT (U/L)	358.40 ± 110.30	375.6 ± 147.8	261.50 ± 115.60	300.00 ± 108.60	410.90 ± 84.05	306.40 ± 39.35	237.60 ± 45.55	0.56

Notes: TP: Total Protein; ALB: Albumin; ALP: Alkaline Phosphatase; GOT: Aspartate Aminotransferase; GPT: Alanine Aminotransferase. Differences among groups were determined using the one-way ANOVA procedure. Means with different lowercase superscripts differ significantly at *p* < 0.05.

**Table 8:** Effect of lysine supplementation in low-protein diets on pelt quality of blue foxes in fur-growing stage.

Items	Lysine						Control	*p* Value
	0.75%	0.95%	1.15%	1.35%	1.55%	1.75%		
Pelt length (cm)	94.50 ± 3.26	96.75 ± 3.79	96.83 ± 3.30	99.88 ± 2.20	97.33 ± 2.94	96.58 ± 4.41	94.67 ± 2.81	0.13
Pelt weight (g)	611.7 ± 30.44	635.0 ± 59.16	637.5 ± 37.65	660.8 ± 48.93	636.7 ± 55.11	651.7 ± 29.10	632.5 ± 32.98	0.60
Guard hair length (mm)	6.74 ± 0.25 ^a^	6.48 ± 0.15 ^ab^	6.28 ± 0.15 ^bc^	6.23 ± 0.16 ^bc^	6.27 ± 0.16 ^bc^	6.22 ± 0.29 ^bc^	6.10 ± 0.11 ^c^	<0.01
Underfur length (mm)	5.08 ± 0.13 ^a^	5.10 ± 0.12 ^a^	5.27 ± 0.18 ^a^	5.38 ± 0.19 ^bc^	5.47 ± 0.16 ^b^	5.42 ± 0.20 ^b^	5.25 ± 0.14 ^a^	<0.01
Guard hair/ underfur	1.32 ± 0.03 ^a^	1.27 ± 0.02 ^b^	1.20 ± 0.02 ^c^	1.16 ± 0.02 ^cd^	1.15 ± 0.02 ^d^	1.15 ± 0.03 ^d^	1.16 ± 0.02 ^cd^	<0.01

Notes: Means with different lowercase superscripts differ significantly at *p* < 0.05.

## Data Availability

The original contributions presented in this study are included in the article. Further inquiries can be directed to the corresponding authors.
